# The role of arginine and arginine-metabolizing enzymes during *Giardia* – host cell interactions *in vitro*

**DOI:** 10.1186/1471-2180-13-256

**Published:** 2013-11-14

**Authors:** Britta Stadelmann, Kurt Hanevik, Mattias K Andersson, Oystein Bruserud, Staffan G Svärd

**Affiliations:** 1 Department of Cell- and Molecular Biology, Uppsala University, BMC, Box 596, Uppsala SE-751 24, Sweden; 2 Institute of Medicine, University of Bergen, Bergen N-5021, Norway; 3 Centre for Tropical Infectious Diseases, Department of Medicine, Haukeland University Hospital, Bergen N-5021, Norway

**Keywords:** Protozoa, Diarrhea, Citrulline, Caco-2, Nitric oxide

## Abstract

**Background:**

Arginine is a conditionally essential amino acid important in growing individuals and under non-homeostatic conditions/disease. Many pathogens interfere with arginine-utilization in host cells, especially nitric oxide (NO) production, by changing the expression of host enzymes involved in arginine metabolism. Here we used human intestinal epithelial cells (IEC) and three different isolates of the protozoan parasite *Giardia intestinalis* to investigate the role of arginine and arginine-metabolizing enzymes during intestinal protozoan infections.

**Results:**

RNA expression analyses of major arginine-metabolizing enzymes revealed the arginine-utilizing pathways in human IECs (differentiated Caco-2 cells) grown *in vitro*. Most genes were constant or down-regulated (e.g. arginase 1 and 2) upon interaction with *Giardia*, whereas inducible NO synthase (iNOS) and ornithine decarboxylase (ODC) were up-regulated within 6 h of infection. *Giardia* was shown to suppress cytokine-induced iNOS expression, thus the parasite has both iNOS inducing and suppressive activities. Giardial arginine consumption suppresses NO production and the NO-degrading parasite protein flavohemoglobin is up-regulated in response to host NO. In addition, the secreted, arginine-consuming giardial enzyme arginine deiminase (GiADI) actively reduces T-cell proliferation *in vitro*. Interestingly, the effects on NO production and T cell proliferation could be reversed by addition of external arginine or citrulline.

**Conclusions:**

*Giardia* affects the host’s arginine metabolism on many different levels. Many of the effects can be reversed by addition of arginine or citrulline, which could be a beneficial supplement in oral rehydration therapy.

## Background

*Giardia intestinalis (a.k.a. G. lamblia and G. duodenalis)*, a protozoan parasite, causes diarrhea in a wide variety of host species [1]. Due to the broad spectrum of hosts and genetic differences the parasite is divided into 8 assemblages (A to H) [2], of which two (A and B) are responsible for approximately 300 million cases of human giardiasis yearly [2]. Giardiasis was included into the WHO initiative for neglected diseases in 2004 [3]. Patients get infected upon ingestion of infectious cysts in contaminated food or water that release proliferating trophozoites in the duodenum, establishing intestinal infection [1]. Roughly half of the infections stay asymptomatic, whereas the other half results in disease. Symptoms of giardiasis include nausea, vomiting, epigastric pain and watery diarrhea [4], though duration and symptoms are highly variable. Giardiasis is associated with malabsorption, reduced growth and developmental retardation in children [5], irritable bowel syndrome, arthritis and chronic fatigue [6]. It is a multifactorial disease but most of the virulence factors remain unknown [2,7].

*G. intestinalis* is able to degrade the amino acid arginine as an energy source via the arginine dihydrolase pathway [8] and two of the enzymes of this pathway, arginine deiminase (ADI) and ornithine carbamoyltransferase (OCT), are released upon *Giardia*-intestinal epithelial cell (IEC) interaction [9]. The parasite rapidly reduces the amount of arginine in the growth medium during *in vitro* growth [7], resulting in reduced proliferation of IECs. Infection of IECs with an assemblage A isolate of *Giardia* leads to a reduction of nitric oxide (NO) production by these cells [10] since arginine is also substrate for NO synthases (NOS). *Giardia* ADI was identified as the protein being responsible for a reduced NO response in *in vitro* interaction setups [9]. At least *in vitro*, NO acts cytostatic against *G. intestinalis* trophozoites and inhibits encystation and excystation [10], the two differentiation processes essential for infection. It plays a role in muscle relaxation and thus in mechanical parasite elimination by peristalsis [11,12]. Therefore reduction of the NO response of the host is in favor of *Giardia* growth. More recently, a NO-detoxifying enzyme (flavohemoglobin) was found in *G. intestinalis*, but its expression status upon host cell interaction has not been addressed yet [13,14]. Therefore it needs to be investigated how exactly *Giardia* interferes with the NO response of human IECs.

In mammalian cells, NO is formed either by NOS (eNOS, NOS3 in endothelial cells, nNOS, NOS1 in neuronal cells and iNOS, NOS2 in epithelial, endothelial and inflammatory cells) through conversion of arginine into citrulline and NO in an oxygen-dependent reaction, or through reduction of nitrite in various oxygen-independent ways [15]. NO has multiple roles in the human body, broadly taken together, as a cellular messenger and as an antimicrobial agent [15,16]. NO reacts with reactive oxygen intermediates, forming antimicrobial substances such as nitrogen dioxide, peroxynitrite, S-nitrosothiols, dinitrogen trioxide and dinitrogen tetroxide that will cause damage in the cell wall, the DNA and the proteins of pathogens and also human cells [16]. However, effects of NO on *Giardia* trophozoites do not seem to be exerted by peroxynitrite [17].

Many pathogens are known to interfere with the host’s arginine metabolism. *Salmonella typhimurium, Mycobacterium tuberculosis*, *Helicobacter pylori, Trypanosoma brucei* and *T. cruzi, Toxoplasma gondii* and *Schistosoma mansoni* are known examples of pathogens that compete with host NOS for their common substrate arginine via up-regulation of host arginases [18,19]. Some microorganisms are even known to consume arginine via their own arginases [18,19]. Thereby pathogens can reduce host NO production and increase polyamine synthesis, which is in favor of pathogen growth and survival. However, within such studies it has neither been addressed what functions arginine-metabolizing enzymes apart from arginase or arginine transporters could play, nor has the direct consumption of arginine, or active detoxification of NO, by a pathogen been taken into account.

As shown in previous microarray studies [20] a variety of chemokines are induced upon *Giardia*-host cell interaction that would be potent in attracting immune cells such as B and T cells, dendritic cells, macrophages, monocytes, mast cells and neutrophils to the intestinal mucosa. For yet unknown reasons, *Giardia*-infection, however, leads only to a small increase in intra-epithelial lymphocytes and little or no mucosal inflammation in human giardiasis patients [2,21]. It has recently been shown that consumption of arginine and production of ammonia via *Giardia* ADI affects the phenotype and cytokine production of dendritic cells [22], but it is not known if arginine depletion affects other immune cells.

In the present study we show effects of the intestinal parasite *Giardia* on the innate and adaptive host immune response by focusing on the parasite’s arginine-consuming ability and the enzyme ADI in particular. Effects on host cell’s NO production, expression of arginine-consuming enzymes and T cell proliferation are shown. We also investigated a NO-detoxification system that the parasite induces NO-dependently upon host cell interaction.

## Results

### Expression of arginine-consuming enzymes in human IECs upon *Giardia* infection

**Figure 1:**
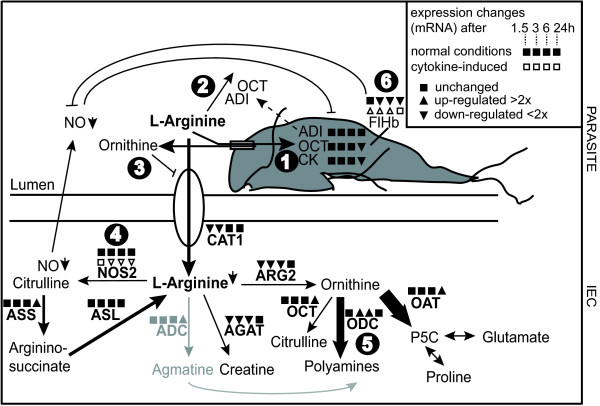
**RNA expression changes of arginine-consuming enzymes upon *Giardia*-host cell interaction.** Based on an interpretation of results from this and previous studies, the encircled numbers point out various ways by which *Giardia* interferes with the host immune response: **(1)** consumption of arginine via arginine-ornithine antiporter, **(2)** release of arginine-consuming ADI and OCT, **(3)** blocking of arginine-uptake into host cells by ornithine, **(4)** down-regulation of host iNOS, **(5)** up-regulation of host ODC, **(6)** up-regulation of parasite FlHb upon NO-stress. Human intestinal epithelial cells (Caco-2) were *in vitro* interacted with *Giardia* trophozoites and the expression changes of arginine-consuming enzymes were assessed by qPCR. Various enzymes involved in the arginine-metabolism of host cells and of *Giardia* are shown (adapted from Stadelmann et al 2012 [7]). Changes in expression after 1.5, 3, 6 and 24 h as compared to 0 h are indicated for interactions with the parasite isolate WB according to Figures 2 and 4 (square for no change, triangle pointing up for up-regulation, triangle pointing down for down-regulation; cut-off value 2). Expression of *inos* and *flhb* in host cells that were stimulated with cytokines (TNF-α (200 ng/mL), IL-1α (200 ng/mL), IFN-γ (500 ng/mL) to produce nitric oxide is also shown (non-filled triangles for up- and down-regulation, non-filled square for no change). ADC, arginine decarboxylase; ADI, arginine deiminase; AGAT, arginine-glycine amidinotransferase; ARG, arginase; ASL, argininosuccinate lyase; ASS, argininosuccinate synthetase; CAT, cationic amino acid transporter; CK, carbamate kinase; FlHb, flavohemoglobin; NO, nitric oxide; NOS, nitric oxide synthase; OAT, ornithine aminotransferase; OCT, ornithine carbamoyl transferase; ODC, ornithine decarboxylase; p6C, Δ1-pyrroline-5-carboxylate.

**Figure 2:**
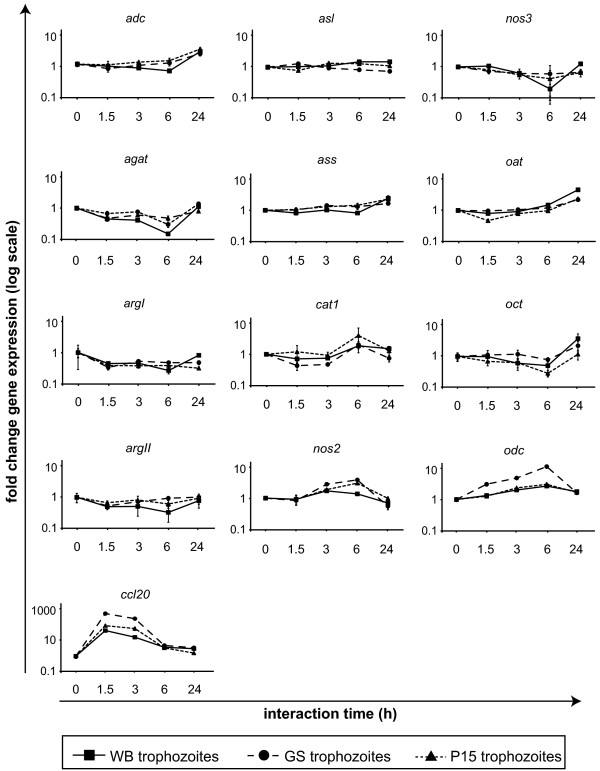
**Expression of arginine-metabolizing enzymes in IECs upon *Giardia *infection.** Differentiated Caco-2 IECs were *in vitro* infected with *Giardia* trophozoites of three different assemblages (isolates WB (squares), GS (circles) and P15 (triangles)) and expression of arginine-consuming enzymes in host cells was assessed after 0, 1.5, 3, 6 and 24 h on the RNA level by qPCR in technical quadruplicates. The data is presented as fold change gene expression in arbitrary units on a logarithmic scale as compared to 0 h expression. GAPDH was used as reference gene. In total 12 different arginine-consuming genes and the control gene *ccl20* were assessed for their expression. Note the changed scale for *ccl20. adc*, arginine decarboxylase; *agat*, arginine-glycine amidinotransferase; *arg*, arginase; *asl*, argininosuccinate lyase; *ass*, argininosuccinate synthetase; *cat*, cationic amino acid transporter; *ccl20*, chemokine (C-C motif) ligand 20; *nos*, nitric oxide synthase; *oat*, ornithine aminotransferase; *oct*, ornithine carbamoyl transferase; *odc*, ornithine decarboxylase.

Our earlier data showed that arginine is depleted in the growth medium already after 1-2 h of *in vitro* interaction between *Giardia* trophozoites and human IECs [7]. A number of enzymes and transporters are directly and indirectly involved in the arginine-metabolism of human cells (Figure 1). Pathogenic microbes are known to affect the expression of these enzymes, especially arginase 1 and 2 [18]. However, arginine-metabolism in human IECs is poorly characterized and it is not known how it is affected by *Giardia* infection. In order to study this, the expression of arginine-consuming enzymes was assessed in differentiated TC7 Caco-2 cells, that exhibit small intestinal epithelial characteristics, by qPCR at time points 0, 1.5, 3, 6 and 24 h post *in vitro Giardia* infection. To study if different *Giardia* assemblages have different effects on the arginine metabolism we used trophozoites from three different isolates: WB (assemblage A), GS (assemblage B) and P15 (assemblage E) [2]. The assessed genes were the chemokine *ccl20* as positive infection control [20] and several arginine-consuming enzymes (see Figure 1 and 2, Additional file 1: Table S1). Except for *cat2* and *nos1*, all tested genes were expressed in IECs, however, *adc*, *argI* and *nos3* only at very low levels (Additional file 1: Tables S2-S4). Most of the genes showed only slight changes in expression on RNA level over the 24 h experiment (Figure 2). The strong induction of *ccl20* already after 1.5 h of infection with *Giardia* trophozoites is in line with our earlier results [20]. None of the tested arginine-consuming enzymes were up-regulated more than 2 times after 1.5 h of WB interaction. After 3 and 6 h, *odc* and *nos2* were up-regulated more than 2 times in the WB interaction, but expression dropped at 24 h. The same observations were made in interactions with parasites of the isolates GS and P15. However, the effects on induction of *ccl20, nos2* and *odc* were much more pronounced upon infection with the isolate GS than with WB and P15 (Figure 2). *arg1, arg2* and *agat* were down-regulated at all time points with a 4- (*arg1*), 3- (*arg2*) and 6.5-fold (*agat*) reduction at 6 h of WB interaction, followed by recovery to near basal expression levels after 24 h. This profile was also seen in interactions with the two other isolates. Several other genes (*adc, oat, oct*) showed the same expression profiles with an initial decrease followed by an increase at 24 h. Thus, upon depletion of arginine by *Giardia* trophozoites (after 1-2 h), expression levels of most host arginine-metabolizing enzymes are reduced, independent of the parasite isolate. The results are summarized in Figure 1, which shows the complex gene expression changes occurring when *Giardia* trophozoites interact with host IECs.

### Effects of *G. intestinalis* on nitric oxide production of human IECs

**Figure 3:**
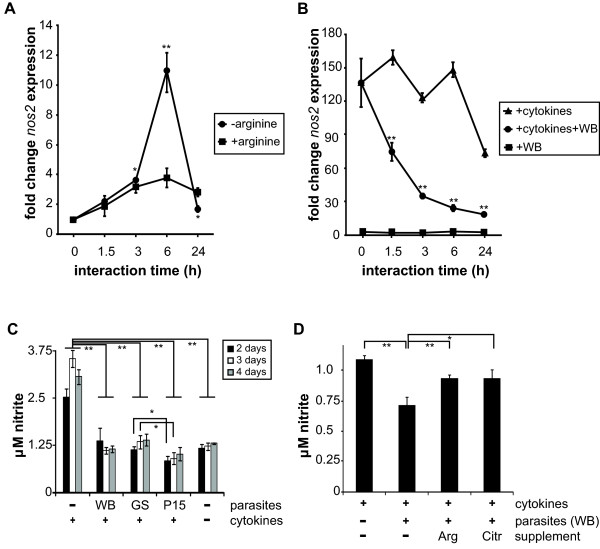
***Giardia *reduces host cell nitric oxide (NO) production. A**, Expression changes of inducible nitric oxide synthase (*nos2)* in differentiated Caco-2 cells in medium with (+ arginine) and without (- arginine) arginine as assessed by qPCR in technical quadruplicates. Data is expressed as fold change expression compared to the 0 h timepoint. Significant expression changes compared to 0 h are indicated by asterisks. **B**, Expression changes of *nos2* upon host cell (HCT-8) stimulation with cytokines (TNF-α (200 ng/mL), IL-1α (200 ng/mL), IFN-γ (500 ng/mL)) and *Giardia* infection 40 h later. Data is expressed as fold change expression compared to the 0 h unstimulated control (squares). **C**, NO production of host cells (HCT-8) stimulated with cytokines 5 h after infection with *Giardia* trophozoites of 3 different isolates (WB, GS, P15). This experiment was repeated two times independently and lead to similar results. **D**, *Giardia* (isolate WB) infected host cells (HCT-8) were stimulated by cytokines to produce NO after 5 h of infection. Subsequently arginine (Arg) or citrulline (Citr) were added to 0.4 mM after 1 h of interaction. NO production was measured 40 h later. The described experiment was repeated two times independently and lead to similar results. Significant differences in the figure are indicated by asterisks (*for p < 0.5 and **for p < 0.01).

Inducible nitric oxide, iNOS, encoded by *nos2*, is a key enzyme in NO production during infections [10,18]. To further investigate the observed effects on the *nos2* expression and iNOS activity in host cells upon *Giardia* infection, effects of different arginine levels were assessed. The growth of IECs in low-arginine medium compared to growth with extra arginine (0.4 mM arginine added to the low-arginine medium) surprisingly showed that *nos2* was highly induced on the RNA level under low-arginine conditions (Figure 3a). The profile of *nos2* induction in low-arginine medium was similar to the profile induced by *Giardia* infection with a peak of expression after 6 h (Figure 2). Strikingly, the level of expression upon parasite-interaction was lower than in the low-arginine medium. We therefore tested the hypothesis that *Giardia* can induce expression of *nos2* via arginine depletion, but at the same time also down-regulate its expression. To test this hypothesis an alternative model was used, where *nos2* expression was first induced in HCT-8 cells by addition of cytokines (TNF-α (200 ng/mL), IL-1α (200 ng/mL, IFN-γ (500 ng/mL) prior to *Giardia* infection (40 h later). Parasite addition clearly and strongly down-regulated the expression of *nos2* (Figure 3b). Thus, *Giardia* can both induce and down-regulate expression of iNOS.

To assess the production of NO upon iNOS induction in *Giardia*-interacted human cells, the NO levels upon infection with isolates of three different assemblages of *Giardia* was assessed. Trophozoites of the isolates WB, GS and P15 were all able to completely suppress NO production of IECs and the IECs did not recover from this within 4 days, even though parasite survival is limited to roughly 24 h within the present interaction system (Figure 3c). Arginine added to physiological concentrations of 0.4 mM could partially restore the NO production of parasite-interacted IECs (Figure 3d). Interestingly, the addition of citrulline, a metabolite of arginine, to a final concentration of 0.4 mM could also restore the capability of IECs to produce NO upon *Giardia* infection (Figure 3d). Thus, *Giardia* can interfere with the innate host immune response by consuming arginine, the substrate of iNOS. Host cells try to compensate this by inducing iNOS, but the parasite can also reduce the expression of iNOS, thereby affecting the host’s NO production.

### Expression of enzymes in *Giardia* upon IEC infection

**Figure 4:**
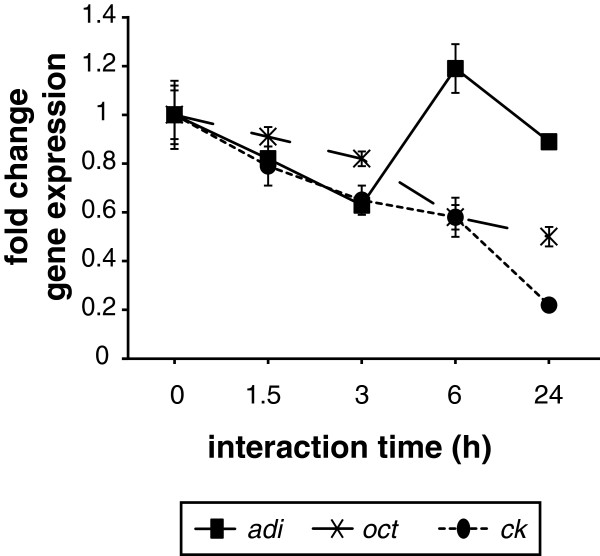
**Expression of arginine-metabolizing enzymes in *Giardia *trophozoites upon host-cell interaction.** Differentiated Caco-2 IECs were infected with *Giardia* trophozoites (isolate WB) and expression of arginine-consuming enzymes (*adi*, arginine deiminase; *oct*, ornithine carbamoyltransferase; *ck*, carbamate kinase) was assessed at 0, 1.5, 3, 6 and 24 h on the RNA level by qPCR in technical quadruplicates. GL50803_17364 was used as reference gene. Fold change gene expression is shown with substracted medium effects.

Apart from expression changes in host IECs, we also assessed the response of *Giardia* enzymes that are directly or indirectly involved in arginine-metabolism upon host-interaction. The three main enzymes of arginine metabolism, ADI, OCT and CK, had previously been shown to be initially up-regulated but later down-regulated after host cell infection [23]. To further investigate this and include also later time points of interaction, trophozoites of the isolate WB were let to interact with differentiated Caco-2 cells for 1.5, 3, 6 and 24 h. Corresponding parasite controls were conducted in host cell medium. Thereby, the parasite genes *adi, oct* and *ck* were down-regulated on the RNA level compared to control samples already after 1.5-3 h (Figure 4, Additional file 1: Table S5). Thus, the down-regulation of the expression of parasite arginine metabolizing enzymes occurs at the same time as arginine is depleted in the growth medium, showing that not only host cells, but also parasite cells, are changing the expression of arginine-consuming enzymes upon interaction.

### Further NO-defending mechanisms of *Giardia*

To test whether the parasite *G. intestinalis* also uses other mechanisms than consuming arginine and changing iNOS expression to combat the antimicrobial host-NO response, the expression of the NO-detoxifying enzyme flavohemoglobin [13,14] (FlHb) was assessed.

**Figure 5:**
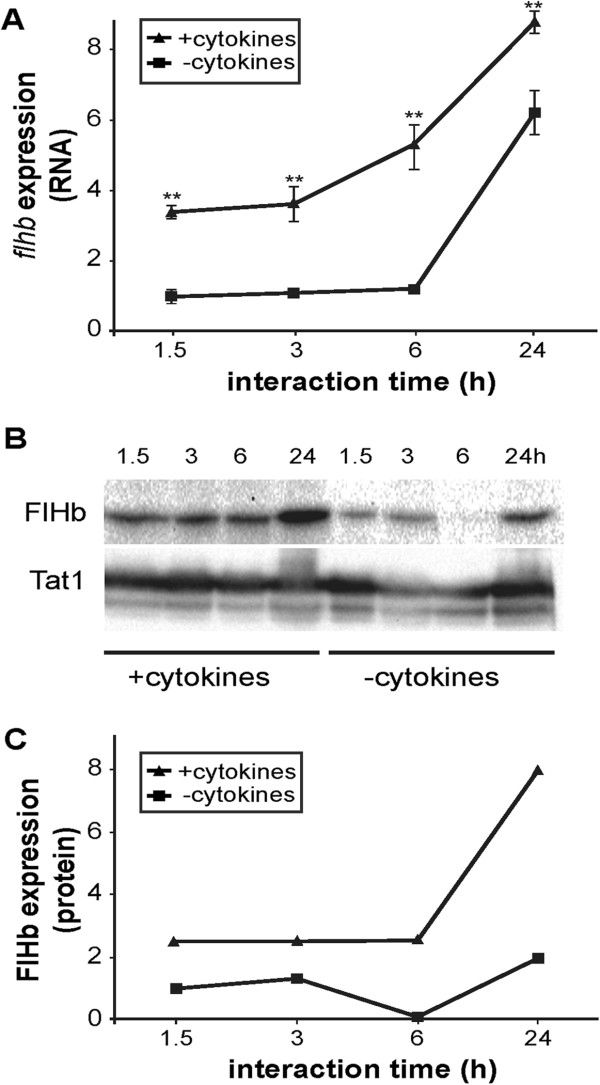
***Giardia *up-regulates flavohemoglobin upon nitric oxide (NO) stress.** Human intestinal epithelial cells (HCT-8) were stimulated for NO production by addition of cytokines (TNF-α (200 ng/mL), IL-1α (200 ng/mL), IFN-γ (500 ng/mL)). *Giardia* trophozoites of the isolate WB were added to the NO-producing host cells and to control cells after 40 h. Samples were measured for expression of the NO-detoxifying protein flavohemoglobin (FlHb) at indicated time points. **A**, Upon interaction with NO-producing cells FlHb was induced in trophozoites on the RNA level compared to the control gene GL50803_17364 as assessed by qPCR in technical quadruplicates. This highly significant difference is indicated by asterisks. **B**, Western blot detecting the expression of FlHb and the control protein Tat1 in *Giardia* upon interaction with HCT-8 cells with and without NO-induction. **C**, Quantification of the Western blot bands (B) by image J software clearly shows the induction of FlHb protein in *Giardia* trophozoites upon interaction with NO-induced host cells. The results are representative for similar results obtained by three independent experiments.

*Giardia* trophozoites were interacted with host IECs that were previously induced to produce NO by addition of cytokines (as described above). Compared to non-stimulated IEC controls, *Giardia* trophozoites up-regulated FlHb expression on the RNA and protein level (Figure 5) when the IECs produced NO. This could provide another layer of NO protection for the parasite (Figure 1).

### Proliferation of arginine-deprived PBMC

**Figure 6:**
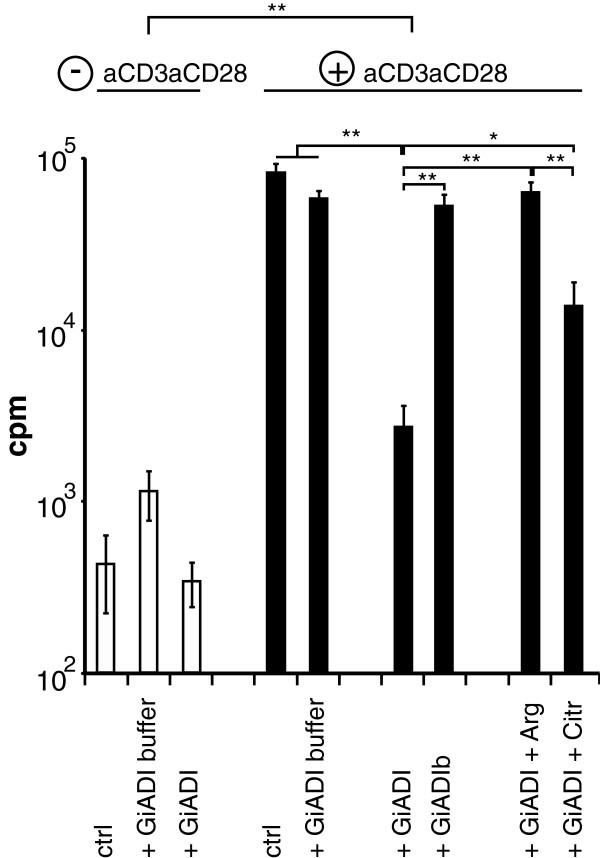
***Giardia *ADI reduces PBMC proliferation through arginine consumption.** The secreted *Giardia* protein ADI (GiADI) was expressed and purified from *Giardia* WB trophozoites. T-cells of human PBMCs of 5 blood donors were stimulated to proliferate by activating anti-CD3 and anti-CD28 (indicated by +) and the proliferation was assessed upon incorporation of ^3^H-labelled thymidine, as depicted in counts per minute (cpm) in logarithmic scale. T-cell stimulation was successful and not inhibited by buffer controls (+ GiADI buffer) or addition of heat denatured GiADI (+ GiADIb). GiADI (5 μg/mL) clearly reduced PBMC proliferation after T-cell specific stimulation, an effect that could be reversed by addition of arginine (+ GiADI + Arg) and partially also by its metabolite citrulline (+ GiADI + Citr). Significant differences are indicated by * (p < 0.5) and ** (p < 0.01).

To assess effects of the local arginine-deprivation caused by *Giardia* on infiltrating lymphocytes, peripheral blood mononuclear cells (PBMCs) were incubated in a concentration series of GiADI and stimulated by T cell activating anti-CD3 and anti-CD28 antibodies. The GiADI used for this experiment was produced in and purified from *Giardia* trophozoites and exhibited *in vitro* arginine-degrading activity as earlier described [7]. There was a dose-dependent repression of T-cell specific PBMC proliferation upon addition of GiADI to PBMCs that reached full effect at 5 μg/mL GiADI (data not shown). This GiADI-dependent repression of PBMC proliferation after T-cell specific stimulation could be reduced by the addition of arginine to 0.4 mM, and partially also by citrulline to 0.4 mM (Figure 6). Respective buffer and denatured protein controls showed no significant inhibitory effects (Figure 6).

## Discussion

The fact that *Giardia* consumes arginine as an energy source is well-known [8,24]. However, possible roles of arginine in the pathophysiology of the host have only recently caught attention [2,7]. Within the present study we therefore assessed the effects of *Giardia*-infection of human IECs on the expression of arginine-metabolizing enzymes. Since gene expression changes during the very first hours of infection can only be studied *in vitro*, we used the *in vitro* interaction system described in [2,7]. We focused on changes on the RNA level since we earlier identified large changes in host cell gene expression already after 1.5 h [20] and early changes of gene expression are best detectable on the RNA level. As shown in Figure 2, most of the host arginine-metabolizing genes were unaffected or slightly down-regulated upon *Giardia*-infection. *nos2,* the inducible form of the nitric oxide synthases (iNOS), was induced after 3 and 6 h of parasite interaction, but down-regulated after 24 h to levels slightly lower than before interaction. We detected a similar induction of *nos2* expression in IECs cultivated without arginine as compared to cells grown with arginine, peaking at 6 h (Figure 3). When we induced iNOS expression in host IECs by addition of cytokines, *Giardia* trophozoites immediately down-regulated this expression (Figure 3), which is not in accordance with earlier results [10], however, fewer parasites per IEC, a different cell line (HT-29), different cytokine concentrations and another experimental approach with measurements after 18 h was used in that study. Thus, *Giardia* infection on one hand immediately induces iNOS by arginine-depletion, but at the same time there are also iNOS down-regulating mechanisms in the parasite. Accordingly, iNOS expression was down-regulated in *Giardia*-infected calves *in vivo* on RNA and protein level after several weeks of infection [25,26].

As shown in Figure 2, the host’s cationic amino acid transporter 1 (CAT1), used for arginine-uptake into host cells, was down-regulated in an early response (1.5-3 h), but up-regulated after 6 h of interaction. This response of co-induction of *nos2* and *cat1*, combined with a down-regulation of arginases, ensures that the host cells take up sufficient arginine for NO synthesis (Figure 1). Such a co-induction has been shown in murine macrophages [27] though it was claimed that CAT2 and not CAT1 is up-regulated together with iNOS [28]. Within our study we could not detect expression of *cat2* in IECs.

A variety of microbes are known to affect the host’s immune response by down-regulating host NO production, either via an up-regulation of host arginases or expression of their own arginases [18,19] that compete for consumption of arginine with iNOS. As shown in Figure 2, host arginases were not up-regulated upon IEC-*Giardia* interaction *in vitro*. However, later time points than 24 h were not included due to limitations of the setup. Whether arginase expression is up-regulated at later time points *in vivo* is, to the best of our knowledge, unknown. Interestingly however, the expression of ODC, a downstream enzyme of arginase, was highly up-regulated at all times (Figure 2). This might lead to a shift of the arginine-flux away from iNOS into polyamine synthesis [7].

*Giardia* infection leads to an increased expression of *odc, inos* and *cat1* during the first hours of interaction, whereas other arginine-consuming enzymes are down-regulated or constant. We therefore studied how the parasite can defend itself against this initial response. As shown in Figure 3, we were able to see a NO reduction similar to *Giardia*-infection of IECs [10] and addition of *Giardia* ADI expressed in *E. coli*[9]. Moreover, this effect was observed for parasites of 3 different isolates (from humans (WB and GS) and pigs (P15)). Interestingly, the observed effect could be reverted by addition of arginine and also by its metabolite citrulline. This finding is interesting with regards to use of citrulline as a supplement in rehydration therapy, as discussed below.

In addition to actively taking up arginine, *Giardia* consumes arginine also indirectly via the secretion of the enzymes ADI and OCT that degrade arginine to ornithine via citrulline [9]. Ornithine, secreted as a final product of arginine fermentation via an arginine-ornithine antiporter [29], has been shown to block arginine transport into IECs [30] (Figure 1). Upon interaction with host cells, the expression of arginine-consuming enzymes ADI, OCT and CK was down-regulated already after 1.5 h on the RNA level (Figure 4), which is in accordance to Ringqvist et al [23]. As suggested, the expression of these enzymes might be increased shortly after secretion (15 minutes after host-parasite interaction), but is down-regulated at later time points due to depletion of arginine in the medium and due to a possible switch to glucose as main energy source [7].

It is not known to date, whether *Giardia* leads to a systemic arginine-deficiency in patients, this needs to be followed up. However, the local reduction of arginine levels by *G. intestinalis* could have additional consequences on the host response, the immune response in particular, since replication and infiltration of immune cells in the intestine might be blocked. It was recently shown that the phenotype and cytokine production of dendritic cells can be affected by the arginine-metabolizing enzyme ADI from *Giardia*[22]. Even though a variety of cytokines are induced upon *Giardia*-host cell interaction, there is no strong intestinal inflammatory response exerted. Nevertheless, a role of T cells in elimination of *Giardia* infection has been shown by Singer and Nash in mice [31]. A specific T cell proliferative response to *Giardia* proteins in humans has been reported [32] and it has been suggested that ADI can inhibit this response [33]. Indeed, we could show that the secreted *Giardia* protein ADI is capable of reducing the human PBMC proliferative response after T cell specific stimulation (Figure 6) and thereby probably inhibit a strong immune response *in vivo*. Maximum effects were gained with a concentration of 5 μg/mL GiADI or above. This amount of GiADI is reasonable for mimicking the *in vivo* situation, since *Giardia* produces and releases ADI constantly. This finding is also in accordance with the decreased proliferation shown for T cells cultured without L-arginine [34] that was shown to be due to down-regulation of the CD3zeta chain of the T cell receptor. Furthermore, we were able to completely revert the observed reduction in T cell specific stimulated PBMC proliferation by addition of arginine to physiological levels (Figure 6).

Arginine is part of certain oral rehydration formulations used for treating diarrhea. However, adverse reactions such as osmotic diarrhea and excessive liver urea production [35,36] are not in favor of such a therapy. In addition, arginine supplementation therapy might also be beneficial for the growth of *Giardia* itself, since the parasite uses arginine as an energy source. For these reasons we also tested the arginine-metabolite citrulline as an alternative supplementary therapy within this study. Citrulline can be reverted into arginine by argininosuccinate synthetase (ASS) and argininosuccinate lyase (ASL), which were both expressed in the IECs used for this study, but not in *Giardia*. It is not clear up to now if citrulline can also be reconverted into arginine *in vivo* by human cells such as IECs, dendritic cells and T cells. However, in children up to 3 years the arginine-reconverting enzymes ASS and ASL are actively expressed in IECs [37]. In addition, ASS and ASL were detected in the canine intestine [38] and it was shown that citrulline supplementation leads to increased arginine levels also in IECs in adult mice [39]. Thus it is likely, that citrulline conversion into arginine is possible in the intestine of human adults. In accordance to this, we could show that citrulline is capable of reversing all the described arginine-dependent effects on NO-production and T cell proliferation that *Giardia* is exerting (Figures 3d and Figure 6). Interestingly, the arginine-dependent block of proliferation that was shown to be induced in IECs upon *Giardia* infection, could also be reverted by citrulline [7]. Therefore citrulline, an abundant component of watermelon, should gain more attention in the future to be used as a supplement in oral rehydration solutions. It could help generating a proper immune response against *Giardia* and inhibiting pathophysiological effects in the intestinal epithelium that are caused by arginine-consumption of *Giardia*.

## Conclusion

The findings presented here, and earlier data, clearly show that *Giardia* interferes with a proper host immune response of the host intestinal epithelium on the innate and adaptive immunity level by affecting arginine in the intesine on multiple levels (Figure 1). The parasite consumes arginine as an energy source [7,24] and thereby the substrate for NOS [10]*. Giardia* trophozoites release arginine-consuming enzymes ADI and OCT [9] and ornithine that blocks the host cell transporter for arginine uptake [29]. Expression of iNOS is initially induced but reduced by the parasite at later stages of infection. Expression of ODC is also induced, further shifting arginine-flux away from iNOS. Flavohemoglobin expression is induced in *Giardia* early upon NO-stress [13]. Dendritic cell cytokine production [22] and T cell proliferation is affected due to reduced arginine-availability. All the observed effects might not be overwhelmingly strong by themselves, but the sum of them will certainly protect the parasite from the host’s response.

## Methods

### Ethics statement

Individuals contributing peripheral blood mononuclear cells (PBMC) for the study of T cell proliferation gave written consent in a standard form upon registration as blood donors. The study and consent procedure was approved by the Regional Committee for Ethics in Medical Research (REK), Bergen, Norway.

### Reagents and cell culture

If not stated otherwise, all chemicals and reagents were purchased from Sigma Chemical Co, USA. *G. intestinalis* trophozoites (strain WB, clone C6 (ATCC30957), strain GS, clone H7 (ATCC50581) and strain P15 were maintained in *Giardia* growth medium, TYDK, as described in Stadelmann et al [7]. *G. intestinalis* trophozoites were used for interaction with human intestinal epithelial cells (IECs) when reaching confluence. They were washed in PBS twice and counted before dilution in complete DMEM (high-glucose DMEM with 10% fetal bovine serum (Gibco®, Invitrogen, Paisley, UK), 4 mM L-glutamine, 1 × MEM non-essential amino acids, 160 μg/mL streptomycin and 160 U/mL penicillin G) and addition to IECs at indicated numbers. IEC cell lines CaCo-2, clone TC7 and HCT-8 (ATCC CCL-244), were maintained as described in Stadelmann et al [2,7], at 37°C, 5% CO_2_, in humid atmosphere, the same conditions that were applied for interaction experiments.

### *Giardia* – IEC interaction: gene expression

For assessment of gene expression of *G. intestinalis* infected human IECs, Caco-2 cells were cultured in T25 tissue culture flasks 21 days post confluence with medium changes twice per week to allow differentiation [9]. Before addition of parasites, IECs were washed in prewarmed DMEM. Subsequently, 7.1×10^6^ parasites were added to culture flasks. Control bottles contained complete DMEM with parasites only. Samples for RNA extraction were taken after 0, 1.5, 3, 6 and 24 h of interaction. Therefore, parasites were detached on ice for 10 min, supernatant was removed and human IECs were washed twice in cold PBS before being taken up in 1 mL TRIZOL® (Invitrogen) and stored at -20°C until further RNA extraction. To extract parasite RNA and protein, the supernatant of interactions including detached parasites was centrifuged at 500×g, 4°C, for 10 min and taken up in 1 mL TRIZOL®.

To assess the expression status of arginine-consuming enzymes in human IECs as well as parasite genes induced upon interaction, RNA was extracted from each respective interaction sample according to the standard TRIZOL protocol. cDNA was prepared and qPCR performed as described in Stadelmann et al [7]. Primers are given in Additional file 1: Table S1. Human *gapdh* (X01677) and *G. intestinalis* WB ribosomal protein S26 (GL50803_17364) were used as reference genes [7,23]. Host cell gene expression was related to the 0 h expression value. Parasite gene expression was expressed relative to the expression of parasites kept in complete DMEM.

### Gene expression in low-arginine medium

To assess the expression of *nos2* under low arginine-conditions, Caco-2 cells were differentiated as described above in complete DMEM over 21 d in culture flasks, with medium changes twice per week. Thereafter, cells were washed in PBS and the medium was changed to low-arginine medium (RPMI 1640 (with L-glutamine, without arginine, leucine, lysine or phenol red) supplemented with 10% fetal bovine serum, 160 μg/mL streptomycin, 160 U/mL penicillin G, 0.4 mM L-lysine and 0.38 mM L-leucine) or low-arginine medium supplemented with 0.4 mM L-arginine as described in Stadelmann et al, 2012 [7]. Samples for RNA extraction were taken after 0, 1.5, 3, 6 and 24 h and *nos2* expression assessed by qPCR as described above.

### *Giardia* – IEC interaction: nitric oxide production

To compare the amounts of nitric oxide (NO) production upon interaction of IECs with different parasite isolates, 5×10^6^ HCT-8 cells were seeded in T25 tissue culture flasks and grown to pre-confluence for 5 days. 3×10^6^ parasites (isolates WB, GS and P15) were added to each flask, including PBS controls. 5 h later cells were stimulated for NO production by adding cytokines (TNF-α (200 ng/mL), IL-1α (200 ng/mL; Santa Cruz Biotechnology), IFN-γ (500 ng/mL; Santa Cruz Biotechnology)). Supernatants for NO measurement were taken after 2, 3 and 4 days of incubation, centrifuged for 10 min at 500×g and supernatants stored at – 20°C until measurement. Therefore triplicates of each sample were measured. 100 μL of the supernatant was reduced by 100 μL of nitrate reductase mix including 0.06 U/mL nitrate reductase from *Arabidopsis thaliana*, 2.5 μM FAD and 100 μM NADPH in K_2_HPO_4_ (50 mM, pH 7.5) in 96 well plates, for 3 h at 37°C. Subsequently, nitrite was detected by the Griess Reagent System (Promega, Madison, WI) according to manufacturer’s instructions. The absorbance at 540 nm was read in a Multiskan MS Plate Reader and nitrite concentrations were calculated according to a standard curve. To revert the parasite induced effects on NO production, arginine or citrulline were added to 0.4 mM final concentration in the same setup after 1 h of interaction between HCT cells and WB parasites. Supernatants for NO measurement were taken after 40 h of incubation and prepared and measured accordingly.

### *Giardia*-IEC interaction upon iNOS induction: gene expression

In order to assess gene and protein expression changes in parasite trophozoites upon host-cell induced NO-stress, HCT-8 cells were seeded in T25 culture flasks and cultivated and stimulated for NO-production with cytokines as described above. After 40 h, parasites were added to 7×10^6^ parasites per bottle. Host cells and interacted parasites were harvested after 0, 1.5, 3, 6 and 24 h. As controls, samples were also taken from host cells that were stimulated with cytokines but not interacted with parasites, or not stimulated with cytokines but interacted with parasites for the same time intervals. To assess the expression of *inos* in CaCo-2 cells, these were taken up in 1 mL TRIZOL® for further RNA extraction and qPCR as described above. Parasites were taken up in 1 mL TRIZOL® for subsequent RNA and protein extraction. cDNA synthesis and qPCR were performed as described above.

To assess expression status of *Giardia* flavohemoglobin also on protein level, Western blot was performed. Protein from interaction setups was extracted from TRIZOL samples and Western blot performed by blocking of protein-containing BioTraceTM PVDF membrane (Pall Corporation, Pensacola, FL) in 3% non-fat milk in PBST. Proteins were detected by use of rabbit anti-*Giardia*-flavohemoglobin (by courtesy of Alessandro Giuffrè, University of Rome, Italy) 1:5’000 diluted in 0.3% non-fat milk in PBST including also a loading control (mouse monoclonal Tat1, 1:5,000 [40]). Secondary HRP-labeled antibodies anti-rabbit and anti-mouse were diluted 1:8,000 and 1:10,000 respectively in 0.3% non-fat milk in PBST. HRP was detected using Western Lightning® ECL Pro (PerkinElmer Inc, Waltham, MA USA) and chemoluminescence detected in a Universal Hood III (Bio Rad). Semi-quantitative comparison of bands was performed by ImageJ 1.32j.

### PBMC acquisition and culture

Peripheral blood mononuclear cells (PBMCs) were isolated by density gradient separation using Lymphoprep (Axis-Shield, Oslo, Norway) from buffycoats obtained from 5 healthy blood donors after routine blood donation. PBMC were washed in NaCl before cells were dissolved in X-vivo 15 serum-free culture medium supplemented with L-glutamine, gentamicin and phenol red (BioWhittaker, Walkersville, MA, USA). The freshly isolated PBMC (8×10^4^ cells/well; 200 μl medium/well) were cultured in 96-well U-bottom microtiter plates in X-vivo 15 medium. Cells were cultured in medium alone, or in the presence of intact functional GiADI (produced, purified and tested as described in Jerlstrom-Hultqvist et al [41]), heat denatured (80°C for 10 min) GiADI (GiADIb), as well as an equal dilution of BSA 1 μg/mL and PreScission enzyme containing buffer used for elution of GiADI, in combinations with 0.4 mM arginine or citrulline and T-cell stimulatory anti-CD3 (mouse IgE moab; CLB-T3/4.E; final concentration 0.3 μg/mL) and anti-CD28 (mouse IgG1moab; CLB-CD28/1; final concentration 0.8 μg/mL) from the Central Laboratory of the Netherlands Red Cross Blood Transfusion Services (Amsterdam, The Netherlands). Cultures were performed in triplicates for 6 days at 37°C in a humidified atmosphere of 5% CO_2_.

### PBMC proliferation assay

Cellular proliferative responses were measured by the incorporation of ^3^H-thymidine into newly synthesized DNA by conventional proliferation assay [42]. After 5 days of culture cells were pulsed with 37 kBq/well of ^3^H-thymidine (Perkin Elmer, Boston, MA, USA) and harvested 18 h later onto glass-fibre pads. Amounts of DNA-incorporated radioactivity were determined by liquid scintillation counting. Proliferation was determined as counts per minute (cpm).

### Data analysis

If not mentioned otherwise, all data were analyzed using Microsoft Office Excel 2010. Figures were prepared in Adobe Illustrator CS4. Statistical analyses were performed by two-tailed student’s t-test (p-value <0.5, significant; < 0.01, highly significant).

## Competing interests

The authors declare that they have no competing interests.

## Authors’ contributions

BS planned and performed all experiments, except the T cell proliferation study, and wrote the manuscript. KH and OB performed the T cell study. MA performed the NO reduction experiment. SGS conceived the study, participated in its design and wrote the final version of the manuscript. All authors read and approved the final manuscript.

## Supplementary Material

Additional file 1 **Describes primers used in RT-PCR analyses (Table S1), expressions of arginine consuming enzymes in IECs interacting with strain WB (Table S2), GS (Table S3) and P15 (Table S4).** Table S5 describes expression of arginine-consuming enzymes in Giardia WB trophozoites during interaction with IECs. Click here for file
